# Bad Breath

**Published:** 1860-10

**Authors:** 


*Bad Breath.*—The following recipe is taken from an exchange: Chlorate of potash, two ounces; sweetened water four ounces; a teaspoonful to be taken a few hours after breakfast, and the mouth washed occasionally with it. This trouble principally arises from a diseased condition of the gums, often produced by secretion of calculi around the necks of the teeth; when dependent upon this cause, all correcting agents will be but temporary. The only cure is the removal of all accumulations, and restoring the gums to health by frequent friction, as from a brush with astringent washes containing subborate of soda. B.

